# AI-Driven Electrical Fast Transient Suppression for Enhanced Electromagnetic Interference Immunity in Inductive Smart Proximity Sensors

**DOI:** 10.3390/s24227372

**Published:** 2024-11-19

**Authors:** Silvia Giangaspero, Gianluca Nicchiotti, Philippe Venier, Laurent Genilloud, Lorenzo Pirrami

**Affiliations:** 1 iSIS Institute, HEIA-FR, HES-SO University of Applied Sciences and Arts Western Switzerland, 1700 Fribourg, Switzerland; 2 Contrinex SA, Route du Pâqui 3, 1720 Corminboeuf, Switzerland; philippe.venier@contrinex.com (P.V.); laurent.genilloud@contrinex.com (L.G.)

**Keywords:** signal denoising, deep neural networks, convolutional neural networks, recurrent neural networks, long short-term memory, gated recurrent unit, compression techniques, EMI, inductive smart proximity sensors

## Abstract

Inductive proximity sensors are relevant in position-sensing applications in many industries but, in order to be used in harsh industrial environments, they need to be immune to electromagnetic interference (EMI). The use of conventional filters to mitigate these perturbations often compromises signal bandwidth, ranging from 100 Hz to 1.6 kHz. We have exploited recent advances in the field of artificial intelligence (AI) to study the ability of neural networks (NNs) to automatically filter out EMI features. This study offers an analysis and comparison of possible NN models (a 1D convolutional NN, a recurrent NN, and a hybrid convolutional and recurrent approach) for denoising EMI-perturbed signals and proposes a final model, which is based on gated recurrent unit (GRU) layers. This network is compressed and optimised to meet memory requirements, so that in future developments it could be implemented in application-specific integrated circuits (ASICs) for inductive sensors. The final RNN manages to reduce noise by 70% (MSE_red_) while occupying 2 KB of memory.

## 1. Introduction

Inductive proximity sensors are key in position-sensing applications in almost any industry, where they detect the presence of metallic objects without physical contact. These sensors create a magnetic field (MF) that, once interacted with by a metal target, induces an eddy current in the object due to electromagnetic induction. Moving the object closer to the sensor causes the current to increase. As a result, the load on the oscillator increases and the MF decreases. Such an amplitude change is detected by the sensor and gives out a signal, allowing the distance of the target to be judged.

However, in order to be used in a harsh industrial environment, inductive sensors must be immune to electromagnetic interference (EMI). The immunity is verified by standardised tests according to the International Electrotechnical Commission (IEC) 61000-4-4 [1] (electrical fast transients/bursts) and the IEC 61000-4-6 [2] (conducted disturbances, induced by radio-frequency fields).

The usual approaches for improving the EMI immunity in inductive proximity sensors are shielding the electronics from the sensor housing, enhancing the decoupling of sensor supplies, and filtering disturbances. However, it is difficult to implement conventional low-pass and band-pass filters without reducing the signal bandwidth, which is between 100 Hz and 1.6 kHz depending on the model of the sensor. In this study, a novel approach was developed to implement a lightweight, compressed deep neural network (DNN) to denoise inductive sensors’ signals from EMI perturbations. This approach makes it possible to exploit the ability of deep learning (DL) to automatically remove EMI characteristics.

Recent advances in the application of artificial intelligence (AI) methodologies provide encouraging results in EMI detection and mitigation. Wang et al. examined the efficiency of convolutional neural networks (CNNs) in predicting and mitigating EMI from the output data of a weather station radar receiver. They had found that deep CNNs can be effective in EMI detection and quality improvement [3]. The study of EMI affecting the instructions of an embedded system was also introduced by Yuan et al., who showed the capability of deep CNNs in classifying different combinations of EMI instructions that embedded systems emit when executing different programs with a prediction accuracy of 83% [4]. In line with these advances, Mitiche et al. developed a fault detection system for EMI power asset diagnostics within the framework of structural health monitoring. The system used a 1D-CNN that handled the one-dimensional data of a high-frequency current transformer and guaranteed a remarkable performance in real-time fault detection [5].

However, although DL is widely used for signal denoising [3,6,7], it often involves the development of very complex computational models, and this poses challenges in resource-constrained hardware implementation, such as application-specific integrated circuits (ASICs). Nevertheless, lightweight NNs have proven valuable for signal feature extraction in many areas [8]. As the ultimate goal is to implement the NN in ASIC for smart inductive proximity sensors, the purpose of this preliminary study is to develop a DNN that meets the study’s objectives, and to compress and optimise it to guarantee the best tradeoff between denoising performance and hardware impact.

**Figure 1:**
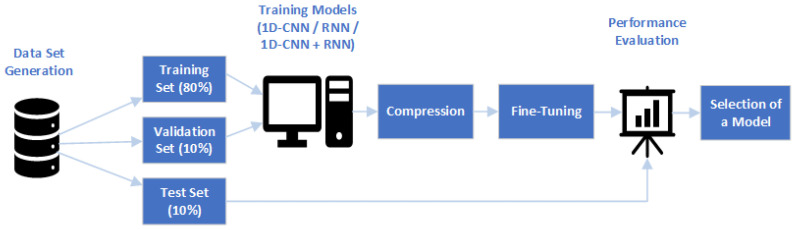
Overview of the main stages of the study.

The main contributions of this paper are as follows (Figure 1):

- Generation of a data set consisting of 1D signals corresponding to inductive proximity sensors’ outputs corrupted by high-frequency perturbations.
- Definition of different NN models for denoising the signals. The architecture and hyperparameters of the networks were optimised in order to achieve the best possible performance.
- Definition of a workflow to further optimise and compress the NNs to minimise their impacts on the hardware’s memory needs. Neurons’ activations were analysed using principal component analysis (PCA), a layer projection technique, to identify and maintain only the essential components of neuronal activations. However, due to dimensionality reduction, this technique caused a loss of information and, thus, a reduction in the performance. For this reason, the projected NNs were retrained to obtain fine-tuned NNs that yield a similar accuracy to the original networks.
- Comparison of NNs, considering two key metrics: performance and network size.
- Identification of the most suitable model for the task.

The rest of the paper is organised as follows. Section 2 reviews some related literature and analyses DL approaches to similar problems. Section 3 describes the method presented in detail. Section 4 presents the performance of the different NN approaches and the results of the compression and optimisation process. Section 5 summarises our considerations and future developments of the research topic.

## 2. Related Work

In order to identify which possible DL strategies could be used for the task at hand, a state-of-the-art analysis was carried out, focusing on studies that used DNNs for both classification and regression goals on time series inputs.

Most studies use CNNs, which have achieved remarkable results especially in the fields of object detection and image classification [3]. With their structure consisting of alternating convolutional and pooling layers, they are able to extract spatial and depth features from the input data. Some studies have employed 2D-CNNs, which transform one-dimensional data into two-dimensional formats to extract and exploit features in both the time and frequency domains [3,4,7,9]. Generally, to generate images from time series, the squared magnitude of the short-time Fourier transform (STFT) of the signals is calculated to form the spectrogram, i.e., a visual representation of the frequency spectrum as it varies with time. Since CNNs were created and modelled to be suitable for 2D data, other methodologies focus on directly using raw time series signals through the development of 1D-CNNs [5,10,11,12,13]. The main advantage is that 1D-CNNs guarantee easier hardware implementation for real-time and low-cost applications, since they use 1D convolutions (scalar multiplications and additions). Indeed, if we consider a 2D-CNN and a 1D-CNN with the same configuration, the same architecture, and the same hyperparameters, and consider a KxK kernel and an image of size NxN, their complexities are respectively $∼O(N^{2}K^{2})$ and ∼O(NK) [14]. Although 1D-CNN is a more suitable model for handling time series, in general, convolutional models were not made for temporal sequence processing. The path commonly taken for sequential data processing is that of recurrent neural networks (RNNs), as they process data in multiple time steps, instead of processing them in a single time step (as in feedforward networks). The standard architecture of RNNs is based on long short-term memory (LSTM) layers, which exploit a mechanism of gates that control the flow of information into and out of the network. LSTM layers are generally used for time series, speech, and text with long-term dependencies [15]. Olickal and Jose use an LSTM-based network as a state estimator for an orthogonal frequency division modulation wireless communication system [16], Do et al. for the detection of vibrational anomalies [17], Liu et al. for the diagnosis of rotating machinery faults [18], and Marchi et al. for acoustic novelty detection [19]. Like the LSTM, the gated recurrent unit (GRU) network is also used to solve difficulties related to limited memory capacity, enabling the model to remember relevant information about the signals for longer periods. It is a very similar but simplified version of the LSTM, where, instead of having three gates (input, output, and forget gates) as in the LSTM NNs, there are only two, the reset and update gates [20]. GRU networks are therefore lighter than LSTM NNs because they have fewer learning parameters and seem to work better on less complex sequences, whereas LSTM layers are better able to process more complex data [21].

Some studies use a mixed approach consisting of combining convolutional blocks with recurrent layers [6,22]. As evidenced by Obeidat et al. (who used the hybrid approach for an automated beat-wise electrocardiogram classification system [22]) and Zhang et al. (who used it to denoise laser Doppler velocimeter signals [6]), combining convolutional layers with recurrent layers allows leveraging the feature extraction capability of CNNs and the advanced temporal sequence processing abilities of RNNs, making it possible to construct a shallower yet effective network.

In this study, we explored the different opportunities identified in the literature and developed three DNN architectures: a 1D-CNN, an RNN, and a hybrid network given by the combination of convolutional and recurrent layers. We have ruled out the analysis of a 2D convolutional approach to compare the three models in the most unbiased manner (with the same mode in which the temporal sequence is given as input to the models) so that the pre- and post-processing steps of the 1D data in 2D can be avoided in a future hardware implementation.

## 3. Materials and Methods

### 3.1. Production of Data Set

Inductive smart proximity sensors from Contrinex SA were used as a reference for the data collection. These sensors are available in different sizes and with measurement distances between 1.5 and 40 mm. Inductive sensors have a signal bandwidth ranging between 100 Hz and 1.6 kHz, depending on sensor size: the M30 model has a bandwidth of 100 Hz and the M8 model of 1.6 kHz.

To train the NNs, it was necessary to have a representative data set of signals corrupted by electrical fast transient/bursts due to EMI, as well as their corresponding clean signals. These signals represent, respectively, the predictors, thus the inputs of the network, and the targets, thus the continuous variables that should be predicted through the network.

The IEC 61000-4-4 [1] is the standard that defines the rules for performing the repetitive fast transient test, which is essential to assess the immunity of electrical and electronic equipment to high-frequency perturbations. The setup mandated by the IEC 61000-4-4 does not allow the voltage signal of the sensor to be measured as the object moves. The difficulty in obtaining real numerical data arises from the fact that, when making measurements directly on the corrupted signal, at the output of the analogue front end, the measurement itself perturbs the signal. Moreover, also creating a sufficiently accurate mathematical model that describes sensor signals proved to be complex.

**Figure 2:**
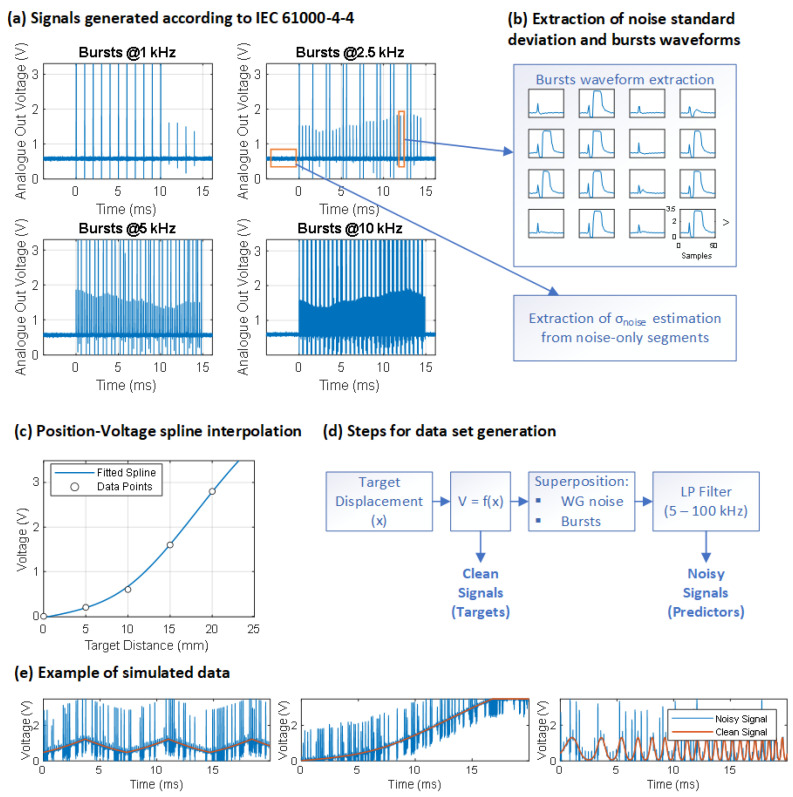
Data set production procedure. (**a**) Signals generated according to IEC 61000-4-4 [1], representative of the analogue voltage output to Contrinex M30 inductive sensors, disturbed in 15 ms windows by bursts at frequencies of 1 kHz, 2.5 kHz, 5 kHz, and 10 kHz, respectively. (**b**) Extraction of noise and burst information from real signals: to obtain an estimate of the amount of noise in the signals, the standard deviation ($\sigma_{\mathrm{noise}}$) of the four signals in the window prior to the start of burst generation was extracted. The average $\sigma_{\mathrm{noise}}$ value of the four signals was taken as a reference value for the simulated signals. The waveforms of the bursts were extracted by moving a window on the signals. (**c**) The voltage (V) as a function of the displacement of the target (x) was calculated according to the response diagram provided by the data sheet of M30 inductive sensor [23]. (**d**) The diagram describes the procedure used for the simulation of new signals. A signal indicative of x was generated. V was calculated as f(x), where f was found in (**c**). AWGN was added to the clean signal with a σ of ±50% of the reference value found in (**b**). Bursts were added to the signal with a random frequency between 1 and 10 kHz. The signals were finally low-pass filtered at a random frequency between 5 and 100 kHz to simulate more real-world situations where disturbances are more attenuated. The signal thus obtained was the noisy signal. (**e**) Examples of simulated signals, representing, respectively, a target moving forward and backward relative to the sensor, an object moving away, and harmonic motion with an increase in frequency.

To address this, both the data corrupted by perturbations and the ideal data were synthetically generated by leveraging the expertise of Contrinex SA. This process involved mimicking the features and structure of real data, drawing upon specific characteristics from output signals provided by Contrinex SA, which were utilised to test the IEC 61000-4-4. The signals provided were the analogue voltages measuring the sensor–object distance between 0 and 3.5 V and acquired with a sampling frequency of 500 kHz. For testing purposes, the target object was kept stationary, at a fixed distance from the sensor, so the ideal, uncorrupted voltage output would be represented by a flat voltage trend over time. The perturbations were generated through a fast transient/burst generator according to the specifications of IEC 61000-4-4 and the bursts were coupled to the power line of the equipment under test to cause interference on the sensor. The perturbations had a duration of 15 ms and were generated at four different frequencies: 1 kHz, 2.5 kHz, 5 kHz, and 10 kHz. Figure 2a shows the four signals acquired, testing an M30 inductive sensor.

The signals were therefore not indicative of the movement of the target object but could be used for extrapolating useful information, such as the noise present and the shape of the bursts. In order to mimic the voltage output signals, a number of possible target object motions were assumed: linear, uniformly accelerated, constant-frequency and non-constant-frequency harmonic motions, and forward and backward displacements of the target. In the linear motion, the position changed linearly over time with random initial positions and velocities. In the accelerated motion, a quadratic term was added to simulate acceleration with random values. In the harmonic motion, a sinusoidal pattern with random frequency and amplitude was generated. The frequency could also change over time, increasing or decreasing. Finally, a sawtooth wave pattern with random frequency and amplitude represented a forward and backward displacement of the target. This decision was based on the observation of such movements in major industrial sensor applications, such as the detection of metal components on production lines or the estimation of the rotational speed of objects.

The data sheet of the M30 inductive proximity sensor [23] was used to derive the function that relates the voltage output of the sensor to the distance of the object (1). The function f was obtained as a spline interpolation of the points obtained from the data sheet (Figure 2c). The data sheet contained response diagrams for various target object materials. For simplicity, the one for a target object in steel was considered from the various curves available.

(1) $$V_{clean}=f\left(x\right)$$

This function was used to obtain the voltage output due to displacement x of the object and represented the ideal voltage, free of any noise that we would like to obtain (target of the network).

The acquired signals were corrupted by a noise baseline, as can be seen in Figure 2a. To obtain a quantitative estimate of the noise, we extracted the standard deviation information from the signal windows before the arrival of the bursts and used this value as a reference to corrupt the mimicked signals. White Gaussian noise was then added to V_clean_ with a standard deviation of ±50% of the reference value. This assumption is reflected in other studies, in which an additive Gaussian noise (AWGN) was used to model the presence of several noise sources in industrial environments. Iqbal et al. observed that the AWGN model can be used to include the effects of different types of noise due to electrical components, such as thermal noise, flicker noise, and quantisation error [24]. Han et al. emphasised that, even though there are many types of noise distribution in real operations, most of them can take the assumption of being independent of each other; thus, the central limit theorem could be applied, which states that, with an increase in the number of noise sources, the total distribution of noise converges to AWGN [25]. Randomly varying the noise present in the signals allowed us to better differentiate the signals in the data set.

To simulate the effect of EMI on the signals, a window of 50 samples was applied to the acquired signals to extract the disturbance patterns. The window length was chosen to match the duration of the EMI bursts (Figure 2b). To generate the corrupted signals, the collected waveforms were randomly selected and superimposed onto the original signal. The burst frequency was randomised for each signal between 1 kHz and 10 kHz. To simulate EMI situations of varying intensity—more or less critical—the resulting signals were low-pass filtered at a random cut-off frequency between 5 kHz and 100 kHz, which correspond to the two extreme Nyquist frequencies of Contrinex inductive sensors. The final voltage represented the signal corresponding to the simulated movement but corrupted by EMI (predictor of the network).

Figure 2d,e contain the steps performed to mimic the signals and some examples of simulated data.

The data set consisted of 1000 pairs of predictors (noisy signals) and targets (clean signals). All signals had a length of 10,000 samples and a duration of 20 ms. A proportion of 80% of the data set was used for the training set, 10% for the validation set, and the remaining 10% for the test set.

### 3.2. Deep Learning Models

In this section, we discuss the hyperparameter optimisation for the three approaches and describe the different architectures of the implemented DL models.

#### 3.2.1. Hyperparameter Optimisation

To optimise the choice of hyperparameters for the training and for the topography of the three approaches (1D-CNN, RNN, and hybrid 1D-CNN + RNN), the general-purpose hyperparameter tuning library KerasTuner was used [26]. The search space of hyperparameters evaluated for each approach is summarised in Table 1, together with the results obtained for each of them. The best learning rate was sought for all models. Subsequent hyperparameters were defined to optimise the topography of the networks. For the convolutional layers, the optimised parameters were the number of filters in each layer and the kernel size. For the RNN instead, it was investigated whether to use LSTM or GRU layers and how many hidden units to use per layer. For each model, it was also defined whether or not to use a final dropout layer to prevent overfitting by deactivating a fraction of neurons, with a dropout rate of 0.25. For the 1D-CNN approach, it was also defined how many times (whether 1, 2, or 3) to repeat the convolutional blocks (convolutional layer, batch normalisation, and rectified linear unit (ReLU)), for the RNN approach, likewise, it was defined how many times to repeat the recurrent layer and, in the hybrid approach, how many times to repeat the convolutional and recurrent blocks. The architecture of the models will be more thoroughly described in the following sections on the individual approaches.

To run the search, a RandomSearch class tuner was chosen, which investigates random combinations of hyperparameters instead of considering them all to find good parameters more quickly without having to examine the entire parameter hyperspace. The goal of the tuner is to minimise the mean squared error (MSE) regarding the validation data. The maximum number of trials is set to 100 with 1 execution per trial. Each trained model is run for a maximum of 10 epochs, and the training data are split into mini-batches of 32 samples.

#### 3.2.2. 1D-CNN Approach

**Figure 3:**
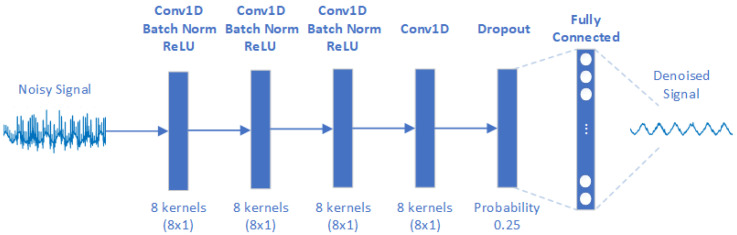
The 1D-CNN approach’s architecture.

In a 1D-CNN network, the input layer takes the time series directly, and convolutional 1D layers apply sliding filters on the input along the time dimension. The architecture consists of four 1D convolutional layers, each consisting of 8 kernels of size 8. These kernels perform convolutions on the signal, processing the raw 1D data and extrapolating features. After each 1D convolutional layer (except the last), a normalisation layer serves to normalise the means and standard deviations of the outputs with the aim of stabilising the learning process and improving model convergence. Next, a ReLU activation layer introduces non-linearity into the model, facilitating the learning of complex functions and improving the network’s ability to predict non-linear relationships inherent in real-world data. Indeed, in the application at hand, noise can affect different frequencies and amplitudes unpredictably, so the use of a ReLU layer offers the possibility of improving the non-linearity characteristics of the problem. After the last convolutional layer, a dropout layer helps the model to generalise and thus reduces the overfitting problem by randomly eliminating certain nodes with a 25% dropout probability. The last layer is a Fully Connected layer that aggregates the features extracted from the previous layers to predict the denoised signal (Figure 3).

#### 3.2.3. RNN Approach

**Figure 4:**
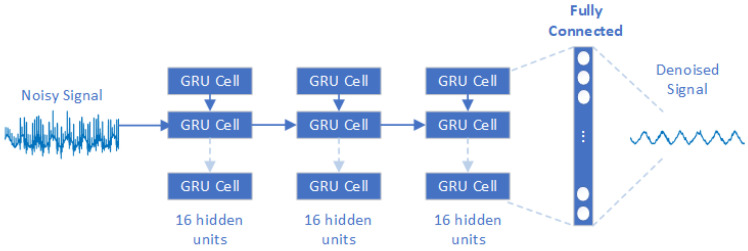
RNN approach’s architecture.

Unlike other DNNs that treat inputs as separate entities, RNNs consider the temporal order of inputs making them suitable for the analysis of temporal sequences [27]. Also, for RNNs, the input signals do not need to be pre-processed, but are directly acquired on the input layer in the time domain, since this type of network is ideal for time series prediction and sequence generation tasks. The most frequently used recurrent layers are the LSTM and GRU layers. LSTM layers use gated units to control the flow of information through the network. They contain three gates: input, output, and forget gates. The gate mechanisms are part of the hidden units, i.e., memory cells that determine the complexity of the model. The number of hidden units therefore determines the size and granularity of the model representation: more units allow the model to store and process more information, also increasing the size of the network. The input gate controls the information in the memory cell, the forget gate controls the information leaving the system, and the output gate controls the process of translating the information into output [20]. A simplified version of LSTM is GRU. It consists of only two gates: the update gate which controls the information entering the memory and the reset gate which controls the information leaving the memory [20]. To decide which approach was most appropriate for the problem at hand, tuning was also carried out by choosing whether to use an LSTM or GRU layer. The result of the tuning reported that the GRU layer performed best. The final model, therefore, has one input layer, followed by three GRU layers, each with 16 hidden units. The architecture ends with a Fully Connected layer that is responsible for the final prediction (Figure 4).

#### 3.2.4. The 1D-CNN-RNN Hybrid Approach

**Figure 5:**
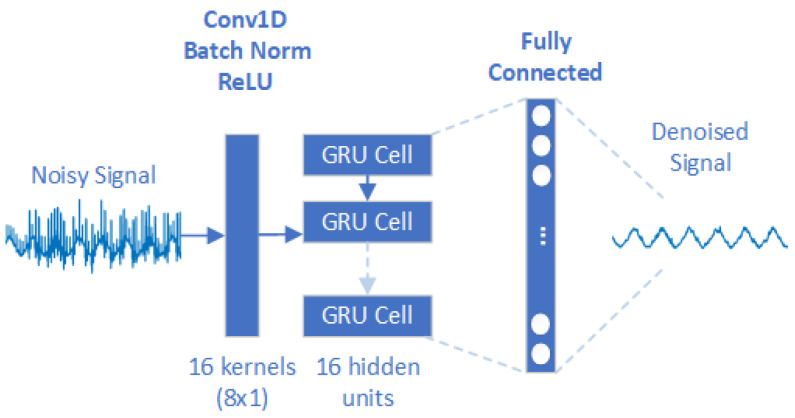
The 1D-CNN-GRU hybrid approach’s architecture.

The last approach combines the advantages of convolutional networks with those of recurrent networks. Noise signals are given directly as input to the network, features are extracted through a 1D convolutional layer consisting of 16 filters with kernels of size 8. The data are then normalised via a normalisation layer, and non-linearity is introduced via a ReLU layer. The data is then introduced into a GRU layer with 16 hidden units and the final denoised signal is formed via a Fully Connected layer (Figure 5).

### 3.3. Training Setting

After identifying the optimal combination of hyperparameters via the KerasTuner, each approach was retrained on 80 epochs with a batch size of 32 samples. An early stopping method was applied with a patience of 10 epochs to prevent overfitting. All approaches used the Adam optimiser to adapt the parameters and the MSE was used as a loss function to quantify how well the model was performing on the training data, by measuring the discrepancy between the prediction and the target. The learning rate was set according to the tuning results, choosing a value of 10^−3^ for the 1D-CNN model and the hybrid model, and a value of 10^−2^ for the RNN model.

### 3.4. Model Evaluation

For this study, we assumed an additive noise model, as often used in methodologies to synthesise noise that replicates sensors’ behaviour [28] and real industrial environment conditions [25]. Noise was directly added to the true signal value to obtain the measured noisy output signal, where the noise denoted the resulting sum of different sources [28]. Thus, the relationship between the noisy and the clean signals can be represented as follows:

(2) u=v+n

where *u* is the noisy signal (input of NN), *v* is the clean signal (target of NN), and *n* is the noise. Thus, the purpose of the networks was to eliminate *n* from the perturbed signals *u*, so that the signal indicative of the motion of the object *v* could be reconstructed. The output of the network was a $\hat{v}$ such that the better the network performed, the closer it was to *v*.

To compare the performance of different models, four evaluation metrics were developed: MSE, MSE reduction (MSE_red_), signal-to-noise ratio improvement (SNR_imp_), and tolerance. The evaluation of the models was carried out on the test set, obtained by extracting 10% of the data set.

(3) $$\mathrm{MSE}={∥\hat{v}-v∥}_{2}^{2}$$

(4) $${\mathrm{MSE}}_{\mathrm{red}}=1-\frac{{∥\hat{v}-v∥}_{2}^{2}}{{∥u-v∥}_{2}^{2}}$$

(5) $${\mathrm{SNR}}_{\mathrm{imp}}=10\log_{10}\frac{{∥u-v∥}_{2}^{2}}{{∥\hat{v}-v∥}_{2}^{2}}$$

(6) $$\mathrm{Tolerance}=\frac{\mathrm{Number}\;\mathrm{of}\;\mathrm{signals}\;\mathrm{within}\;\mathrm{tolerance}}{\mathrm{Test}\;\mathrm{set}\;\mathrm{size}}$$

Smaller values of MSE indicate better denoising capabilities. MSE_red_ indicates by how much, after the application of the NN, the MSE is reduced compared to the initial error (between the clean signal and the noisy signal). SNR_imp_ represents the increase in signal-to-noise ratio after application of the network. The higher the values of MSE_red_ and SNR_imp_, the better the performance of the network. Tolerance represents the number of signals in the test set that respect a certain tolerance. The latter was defined in accordance with the acceptability criteria defined in IEC 60947-5-7 [29], which is the standard regarding the requirements of proximity devices with analogue output. The regulation states that the deviation value of the analogue output signal must not exceed ±10% of the declared output span. Therefore, we thought to identify a metric that would evaluate the difference between the denoised signal samples and the ideal signal samples, and output the number of signals in the test set for which no difference exceeds 10% of the voltage span, which is 3.5 V.

### 3.5. NN’s Compression and Optimisation Method

Most DL models typically work with high-performance processors using millions of learning parameters [30], which complicates implementation in ASICs. In order to be used in small or embedded devices, NNs should be lightweight and compressed.

Compression techniques serve to limit the energy, processing capacity, and storage of DNNs. As noted by Mishra et al. in a systematic review on compression methods, optimisation of the model can be performed in terms of network pruning, sparse representation, bit precision, and knowledge distillation [31]. Optimisation of the model can be achieved by removing filters, connections between neurons, and less fundamental layers (network pruning) or it can be carried out by exploiting the distribution of weights in the models (sparse representation), where the weights establish the importance of connections between specific neurons. Weights that are zero or close to zero can be eliminated, lightening the number of network parameters. It is possible to reduce the accuracy of the weights by using a smaller number of bits, depending on the memory requirements of the model (bit precision). However, when quantising a model, there is a risk of reducing its accuracy. Knowledge distillation techniques therefore transfer information from a “teacher” network to a more compact “student” network, improving its accuracy.

#### 3.5.1. Network Compression Using Projection via PCA

In terms of model pruning, the networks developed in the different approaches were already the result of a compromise between minimising the architectures, reducing the layers, and maintaining performance.

Nevertheless, NNs very often have an abundance of learnables that over-parameterise the task at hand. The network projection approach overcomes this problem, using PCA as a method to reduce the number of parameters while maintaining the most important neuronal connections [30]. PCA seeks to create smaller projected matrices of weights in which only the most significant features and information are preserved. This process involves calculating the eigenvectors of the data covariance matrix, which represent the principal components in which the greatest variance is captured. The eigenvalues indicate the importance of each eigenvector: the greater the eigenvalue, the greater the variance associated with that principal component. Thus, preserving only the most significant eigenvectors, the weights representing the most relevant neuronal connections are retained [32].

Thus, after training the three models, a further reduction in the learnable parameters was achieved using the projection technique. The networks were reduced to meet a specific memory requirement, thus the ReductionGoal was calculated, which is a factor indicating how much the learnables should be reduced in order for the networks to meet the memory requirements:

(7) $$\mathrm{ReductionGoal}=1-\frac{\mathrm{TargetMemorySize}}{\mathrm{MemorySizeOriginal}}$$

Intending in the future to test the network’s denoising ability on the M30 inductive sensor analogue output, and given the memory size constraints present in the hardware, we have decided to set the memory usage limit for the network implementation to 2 KB (TargetMemorySize). MemorySizeOriginal indicates the network size before the projection, and it has been calculated considering that each of the network’s learning parameters is stored on 32 bits:

(8) MemorySizeOriginal=4bytes×numLearnables

The performance on the test set of the resulting networks was then calculated.

#### 3.5.2. Fine-Tuning

**Figure 6:**
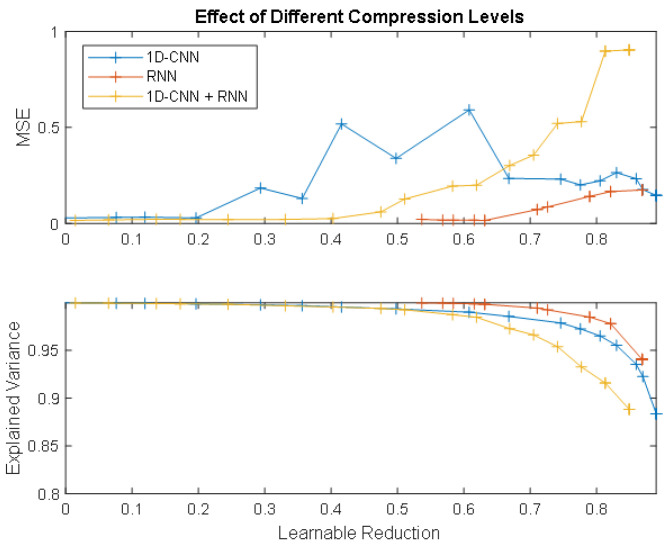
Cumulative explained variance plot for the three approaches.

As fewer and fewer learning parameters are taken into account in the projected models, the explained variance (i.e., the amount of variance regarding the initial total variance that is to be retained [30]) decreases and, along with it, the model error increases (Figure 6). This shows how compressing a model generally results in a degradation of its performance. Consequently, to improve the models, the projected networks were fine-tuned, i.e., the pre-trained projected models were used as a starting point for further training. Generally, a lower learning rate is used to make the process more stable and the training is conducted for fewer epochs. We fine-tuned for 40 epochs, using the Adam optimiser, and reducing the learning rate at $0.5\times 10^{-4}$ for the 1D-CNN and the hybrid approaches, and at $0.5\times 10^{-3}$ for the RNN approach.

Finally, the performance of the fine-tuned projected models on the test set was calculated.

## 4. Results

This section discusses the performance achieved by the different approaches on the test set before and after the compression and fine-tuning procedures, and finally identifies the model that is most suitable for the problem at hand, both in terms of performance on evaluation metrics and in terms of memory.

### 4.1. Comparison Between Original DNNs

Table 2 shows the comparison between the three different original models in terms of evaluation metrics and memory size.

Smaller MSE values and bigger MSE_red_ and SNR_imp_ values show that the RNN model, followed by the hybrid model, achieved the best results in terms of denoising capabilities. The tolerance represents the percentage of signals in the test set which pass the test described by the IEC 60947-5-7 [29]. This metric remains very low in all cases. Only the RNN model manages to remove most of the bursts to such an extent that 24 out of 100 signals fall below the measured threshold. For the hybrid model, the tolerance is lower (10%) and for the 1D-CNN model it is zero. In the latter case, the worst denoising performances are also observed. In this case, the highest value of MSE and the lowest values of MSE_red_ and SNR_imp_ are reflected in an inability of the network to bring the denoised signals below the imposed threshold. In this sense, the tolerance offers a more severe and restrictive analysis than the MSE and SNR, as the threshold of variation from the ideal signal set is very low.

In general, it is observed that all metrics confirm that the recurrent approach is the best. But, in terms of learnable parameters, RNN has significantly more (4049) than 1D-CNN (1689) and 1D-CNN + RNN (1777). This implies that RNNs can represent more complex information, but also require more resources.

### 4.2. Effects of Compression and Optimisation of the Model

As shown in Table 2, following compression, the RNN model remains the one that guarantees the lowest MSE, although, in general, all models show a significant degradation in performance, even negatively impacting in terms of MSE reduction and SNR improvement regarding the baseline. Greater degradation, with the same goal of reducing learnables, implies that the model is less projection compressible and has less redundancy. And this is especially observed in the hybrid approach, which leads to the worst performance.

**Figure 7:**
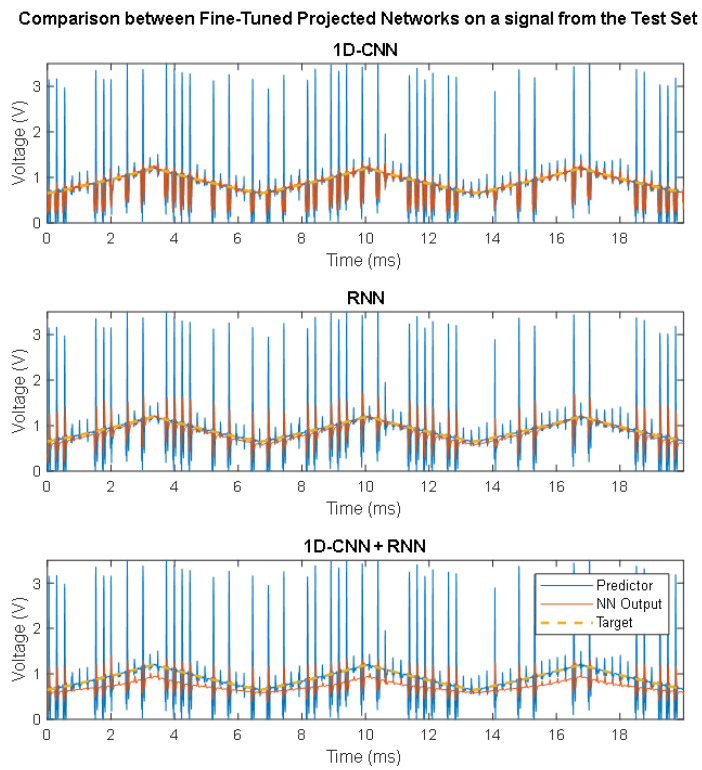
Example of a signal from the test set denoised by the different DNN approaches.

From the compression and optimisation of the models, it would appear that, although after compression the RNN model still performs better than the other two approaches (with an MSE of 0.175 compared to 0.217 for the 1D-CNN and 0.520 for the hybrid model), following fine-tuning, the 1D-CNN manages to recover denoising capabilities better than the RNN. Fine-tuning, thus, seems to have a positive impact on CNNs, but may not be as effective with RNNs. Figure 7 shows the comparison between the three final fine-tuned approaches on a signal extracted from the test set.

Finally, we can conclude that, with an MSE reduction rate of 70% and a memory limited to 2 KB, with only 532 learning parameters, the RNN model is the most suitable for the problem at hand.

## 5. Discussion

The aim of this study is to make a comparison of the most commonly used NNs for processing time series, to identify the one that, given the same memory limits, guarantees the best denoising capabilities. Although several studies seem to show that the combination of convolutional layers with recurrent layers ensures excellent results on the processing of time series as they combine the benefits of the two models (the ability to extract features of CNNs and the processing of temporal sequences of RNNs) [6,22], the results of this study show that, in our case, the use of the simple convolutional or recurrent approach ensures better results.

The model that with only 532 learning parameters and thus 2 KB of memory manages to guarantee a 70% reduction in MSE is the RNN model, consisting of an input layer, which takes the raw data as input over time, three GRU layers and a Fully Connected layer for regressing the output data. The projection technique via PCA can greatly reduce the size of the model by 87%, which is advantageous for resource-limited devices or applications requiring lightweight models. Fine-tuning the models appears to be effective in improving performance compared to the projection alone, but does not fully achieve the capability of the original model.

The RNN is better adapted to the problem at hand, confirming that, in general, recurrent networks are better suited to time series tasks than convolutional networks. The latter, although adapted to the problem, are born for different tasks, concerning image classification and object detection, where their ability to capture local patterns and spatial features is advantageous. Furthermore, between GRU layers and LSTM layers, GRUs emerge as the preferred choice. As pointed out by Cahuantzi et al. [21], GRUs outperform LSTM networks on lower-complexity sequences, while on high-complexity sequences LSTM layers perform better. The problem under consideration, therefore, might be easier from a data complexity perspective and therefore perform better with GRU layers. This leads us to a further advantage in that GRU layers are lighter and require fewer learning parameters.

The network should ideally be customised and re-trained for each sensor to align as closely as possible with its specific parameters. Although our data set so far brings out some features of M30 inductive sensor signals, it may not really reflect all the variation to be expected from real data. For instance, higher sampling rates may result in longer signals, which can pose some challenges with regard to how RNNs can process them [18,27]. The best model, therefore, could be specific to sensor settings. In order to improve the generalisation capability of the network over the whole family of the inductive sensors, it is desirable that a data set be developed that can better represent diversified signals coming from various sensors.

This study represents a first analysis of the state-of-the-art of possible AI solutions for denoising EMI-disturbed signals in challenging environments and under harsh conditions of use. It shows that a lightweight, low-size NN can be developed to improve EMI immunity in conditions where traditional techniques, such as shielding the electronics from the sensor housing, improving the decoupling of sensor supplies, and filtering with traditional filters, are not sufficient. Our ongoing objective is to validate the network’s robustness on real data to assess any potential data set-induced bias. Moreover, future developments will focus on the implementation of the final NN model into Contrinex’s new generation ASICs, that are pivotal in enhancing the performance of smart proximity sensors due to their ability to provide tailored solutions with low-power consumption and compact sizes. We will also explore further enhancements of the network and examine the possibility of quantising single-precision floating point into 8-bit integer data.

## Figures and Tables

**Table 1:** Search space for hyperparameter definitions and their selected values for the three different models.

Model	Hyperparameters	Search Space	Values
1D-CNN	Learning rate	$10^{-5}$, $10^{-4}$, $10^{-3}$, $10^{-2}$	$10^{-3}$
	Number of filters	8, 16, 32	8
	Dimension of kernel	3, 5, 8	8
	Number of layers	1, 2, 3	3
	Dropout	TRUE/FALSE	TRUE
RNN	Learning rate	$10^{-5}$, $10^{-4}$, $10^{-3}$, $10^{-2}$	$10^{-2}$
	Which recurrent layer	LSTM/GRU	GRU
	Number of hidden units	8, 16, 32, 64	16
	Number of layers	1, 2, 3	3
	Dropout	TRUE/FALSE	FALSE
1D-CNN + RNN	Learning rate	$10^{-5}$, $10^{-4}$, $10^{-3}$, $10^{-2}$	$10^{-3}$
	Number of hidden units in GRU	8, 16, 32, 64	16
	Number of filters	8, 16, 32	16
	Dimension of kernel	3, 5, 8	8
	Number of layers (CNN+RNN)	1, 2, 3	1
	Dropout	TRUE/FALSE	FALSE

**Table 2:** Comparison between the different DNN approaches (original, projected, fine-tuned projected) in terms of performance on test set and dimension.

	Performance on Test Set			DNN Dimension		
**Original Networks**	MSE (10^−3^)	MSE (%)	SNR_imp_	Tolerance (%)	Nmb Learnables	Memory Size (KB)
1D-CNN	24.46 ± 16.65	67.03 ± 42.49	7.112 ± 4.336	0	1689	7
RNN	9.104 ± 12.87	84.14 ± 52.88	12.07 ± 4.466	24	4049	16
1D-CNN + GRU	14.14 ± 12.38	76.79 ± 51.99	9.680 ± 4.564	10	1777	7
**Projected Networks**						
1D-CNN	216.8 ± 102.8	−237.8 ± 834.5	−2.911 ± 3.304	0	503	2
RNN	174.5 ± 98.59	−297.6 ± 1385	−1.754 ± 4.320	0	532	2
1D-CNN + GRU	520.4 ± 169.7	−1269 ± 4313	−7.070 ± 4.433	0	460	2
**Fine-Tuned Projected Networks**						
1D-CNN	28.56 ± 16.91	63.65 ± 47.21	6.215 ± 3.775	1	503	2
**RNN**	**29.96 ± 24.78**	**70.35 ± 40.73**	**6.645 ± 2.833**	**6**	**532**	**2**
1D-CNN + GRU	46.19 ± 31.81	27.69 ± 170.4	4.186 ± 3.747	0	460	2

## Data Availability

The data sets presented in this article are not readily available because the data are part of an ongoing project. Requests to access the data sets should be directed to L.P.

## Abbreviations

The following abbreviations are used in this manuscript:

MF	Magnetic Field
EMI	Electromagnetic Interference
IEC	International Electrotechnical Commission
NN	Neural Network
DL	Deep Learning
AI	Artificial Intelligence
DNN	Deep Neural Network
CNN	Convolutional Neural Network
PCA	Principal Component Analysis
ASIC	Application-Specific Integrated Circuit
STFT	Short-time Fourier transform
RNN	Recurrent Neural Network
LSTM	Long Short-term Memory
GRU	Gated Recurrent Unit
ReLU	Rectified Linear Unit
ISFT	Inverse Short-time Fourier Transform
MSE	Mean Squared Error
SNR	Signal-to-Noise Ratio
AWGN	Additive White Gaussian Noise
